# Strategies for Obtaining Obsidian in Pre-European Contact Era New Zealand

**DOI:** 10.1371/journal.pone.0084302

**Published:** 2014-01-08

**Authors:** Mark D. McCoy, Jonathan Carpenter

**Affiliations:** 1 Department of Anthropology & Archaeology, University of Otago, Dunedin, New Zealand; 2 Department of Archaeology & Natural History, Australian National University, Canberra, Australia; University of Oxford, United Kingdom

## Abstract

Archaeological evidence of people's choices regarding how they supply themselves with obsidian through direct access and different types of exchanges gives us insight in to mobility, social networks, and property rights in the distant past. Here we use collections of obsidian artefacts that date to a period of endemic warfare among Maori during New Zealand's Late Period (1500–1769 A.D.) to determine what strategies people engaged in to obtain obsidian, namely (1) collecting raw material directly from a natural source, (2) informal trade and exchange, and (3) formal trade and exchange. These deposits represent a good cross-section of Late Period archaeology, including primary working of raw material at a natural source (Helena Bay), undefended sites where people discarded rubbish and worked obsidian (Bream Head), and a heavily fortified site (Mt. Wellington). We find that most of the obsidian described here was likely obtained directly from natural sources, especially those located on off-shore islands within about 60–70 km of sites. A smaller amount comes from blocks of material transported from an off-shore island a greater distance away, called Mayor Island, in a formal trade and exchange network. This study demonstrates the value of conducting tandem lithic technology and geochemical sourcing studies to understand how people create and maintain social networks during periods of warfare.

## Introduction

The geographic distribution of stone artefacts made of obsidian, a natural volcanic glass, gives us a window in to major shifts in human mobility and trade in the past. Archaeologists have for many years used these spatial patterns to reconstruct changes associated with transition from the Paleolithic to Neolithic which involved increasingly permanent settlement and food production via agriculture [1]. In the more recent past, remarkable social networks that stretched across thousands of kilometers of ocean between Pacific Island communities have been reconstructed based on obsidian evidence [2]. These long-distance connections, generally speaking, broke down over time when natural demographic growth on newly settled islands reached a point when communities were self-sufficient and not as willing to maintain the cost of extreme sea voyaging.

Recently, Walter et al. [3] have argued based on obsidian evidence that there was a shift from ‘settler’ to ‘trader’ motivated interaction among Maori within 300 years of initial human colonisation of New Zealand. Obsidian from the small off-shore island of Tūhua (Mayor Island) is today found in great frequency across the country at sites dated to the first centuries of settlement (1250–1500 A.D), indicating that the ancestors of Maori accessed it directly. However, after 1500 A.D., we no longer see signs of a highly mobile, closely inter-connected series of settler communities [4]. The movement of North Island obsidian is replaced by inter-island trade in objects made from South Island greenstone (jade, Maori: *pounamu*). This coincided with the construction of the first of thousands of earthwork fortification [5], called *pa*, as well as the permanent occupation of Mayor Island [6].

The coincidence between increased evidence for trade, decreased direct access to Mayor Island obsidian, and signs of group-level warfare over property together suggest that people living in New Zealand in the Late Period (1500–1769 A.D.), had a difficult choice to make regarding how they supplied themselves with obsidian. One could directly access the closest source, thereby minimising costs of long-distance travel and/or trade and exchange. Alternatively, one could obtain already extracted obsidian from what is sometimes called down-the-line exchanges, where raw material informally changes hands many times as it travels away from the source area, each time being reduced slightly from its original size as part of it is retained by the previous owner. Of course, given that we have good evidence for trade in greenstone, it is also reasonable to imagine one might also choose to engage in more formal trade and exchange, assuming of course that obsidian was valued enough to continue to be transported long distances.

However, while it may be tempting for social scientists to view these choices in terms of simplistic, rational economic costs and benefits, we learn far more through careful consideration of the emic values of Maori. As ‘people of the land’ (*tangata whenua*), land is not owned in the strict Western sense of the word, rather Maori define group-level and personal identity with reference to specific places on the landscape. This is not to say that land was an unrestricted commons (e.g., *res nullius*), rather it is more precise to say that land was held in common and one could assert one's rights with regard to a specific place through genealogy (*whakapapa*, or genealogical layering). While rights to land are primarily derived from inheritance, they also come with an obligation to manage resources (i.e., customary guardianship, *kaitiakitanga*), and required continued use to maintain rights [7]. The guiding principle of customary guardianship follows a culturally particular interpretation of traditional authority (*mana*), spiritual life-principle (*mauri*), sacredness (*tapu*), prohibition/conservation (*rahui*), hospitality (*manaaki*), and transfer/gift/release (*tuku*), which continues to resists simple translation in to a modern Western legal framework [8]. Gift exchange and trade are no less layered with spiritual value and social obligation. An object (*toanga*) given as a gift (*koha*) implies obligation to reciprocate to achieve the cultural ideal of balance (*utu*), and failure to reciprocate in kind put one in mortal spiritual danger [9].

We present an analysis of strategies people used to supply themselves with obsidian in an effort to determine what was more common among Late Period Maori communities: direct access, informal trade-and-exchange, or formal trade-and-exchange? First individual artefacts are matched to their geological source based on geochemistry. Next, assemblage-scaled lithic technology analysis is used to determine which of these strategies appears to account for the obsidian found. While obtaining obsidian was likely a minor concern when compared with other material needs, such as maintaining a secure supply of food, the value of research like this is it exposes key elements of how an ancient society operated in practice through a direct historical approach. Specifically, the results point to persistent kin networks materialised through the collection of obsidian from natural sources, and a high degree of formality with regard to gifts and trades.

## Materials and Methods

All necessary permits were obtained for the described study, which complied with all relevant regulations. Field research permits for the work described here were obtained from the New Zealand Historic Places Trust (Helena Bay, Authority 2010/392; Bream Head, Authority 2007/97; Mt. Wellington, permission granted prior to modern inventory numbering system). The sites are all located on publicly owned land (Helena Bay, Whangarei District Council; Bream Head, Department of Conservation; Mt. Wellington, Auckland City Council).

### Archaeological Collections

**Figure 1:**
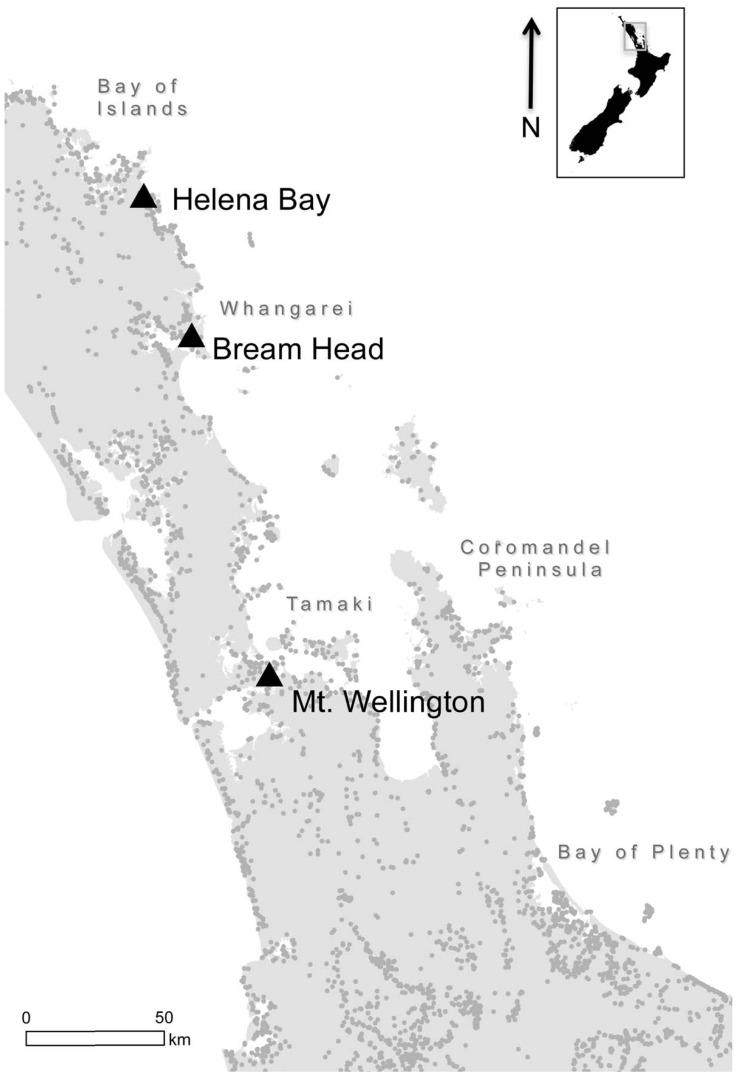
Known Late Period Fortifications in the Study Area.

The obsidian artefact collections examined here were excavated from three locations on the east coast of New Zealand's North Island: Helena Bay (Te Mimiha) just outside the Bay of Islands (Ipipiri) region, Bream Head (Te Whara) at the mouth of the Whangarei Harbour, and at Mt. Wellington (Maungarei) a large volcanic cone on the Auckland Isthmus (Tāmaki) (Fig.1). At the time of regular European contacts in the 19^th^ century these three regions – the Bay of Islands, Whangarei, and Tāmaki – would have been among the most densely populated in the country. The concentration of hillforts and discarded food refuse deposits, referred to as shell middens due to their high marine shell content, suggests this was likely also the case throughout the Late Period.

#### Helena Bay (Te Mimiha), Bay of Islands Region

The first study area, Helena Bay, is a small inlet where the Huruiki Stream meets the Pacific Ocean. It is well within the ‘source area’ of a type of obsidian commonly referred to as the Huruiki source [12]. While the primary geological source of raw material is several kilometers inland, we presume that obsidian cobbles would have also been in the stream and foreshore. The collections examined here come from a deposit located on a consolidated Pleistocene dune behind the foreshore (Q05/567). Based on the large size and extraordinary density of obsidian found, these deposits are interpreted as reflecting quarrying reduction [13]. While a hearth was discovered in these deposits, no material appropriate for radiocarbon dating was identified. Nonetheless, the lack of midden with extinct taxa and the location of the deposit immediately below the turf suggest it likely reflects Late Period activities. The obsidian working area is at the foot of Te Maurea Pa (Q05/322 and 323), which extends for 500 m along the ridgeline above the southern end of the beach.

#### Bream Head (Te Whara), Whangarei Region

Bream Head is a landscape dotted with dense patches of midden along the entryway to the Whangarei Harbour, some of which date back to the Early Period. The material examined here comes from a midden overlying several earlier earth ovens (Feature 1 - Q07/78, n = 10) and obsidian working area (Feature 22 - Q07/774, n = 11) at Urquharts Bay; and an obsidian working area between Urquharts Bay and Frenchmans Bay (Feature 8 - Q07/747, n = 12) [11]. Radiocarbon dating and the lack of extinct taxa put these features squarely within the Late Period. We also note that these deposits are located outside the defences of the nearest *pa*, and thus represent activities taking place in an undefended area.

#### Mt. Wellington (Maungarei), Auckland Region

Lastly, Maungarei (Mt. Wellington) is one of the largest and best studied fortifications in New Zealand. The entire site is given the designation R11/12 and but this refers to series of discrete features, including house sites, terraces, middens, and earthwork defences that were investigated between 1962 and 1970 [10]. Radiocarbon dating of material from across the site indicates the main period of occupation is likely 1580–1660 A.D. [10].

In total, 253 obsidian artefacts are included in this study, with 95 from Helena Bay, 33 from Bream Head, and 125 from Mt. Wellington. These are essentially all the examples of obsidian collected from excavations to date at these sites, with the exception of Helena Bay where a particularly large number of individual pieces have been recovered. The subset of Helena Bay artefacts discussed here in detail (n = 95), is a fraction of those assessed for chemical composition from the site (n = 329).

### Lithic Technology

Individual artefact attributes recorded for the entire obsidian assemblage included standard quantitative and qualitative data [14], specifically: weight (g), length (mm), width (mm), thickness (mm), artefact type (e.g., flake, core), presence of cortex (e.g., weathered natural surface of raw material), number of flake scars, and presence of macroscopic evidence of edge damage from use. Since our main focus was on lithic technology rather than use we employed a conservative approach that represents the minimum frequency of useware. For example, evidence of damage along all the edges of an artefact, a pattern that might have been created by post-depositional processes, were classified as having no edge damage and no effort was made to identify microware or residue representing use.

While is it is impossible to say for certain the way an individual artefact was obtained, the purpose of lithic technology analysis is to make a general assessment of whole assemblages relative to expected patterns left by different behaviours. Direct access for example, because it is associated with regularised trips made to quarry or collect material from the natural source, would be expected to produce a significant number of artefacts with cortex. The most unequivocal evidence for direct access in the archaeological record is of course discrete formal sites of quarrying in close proximity of a natural source. However, because New Zealand obsidian was in most cases collected informally, our best chance of identifying quarrying requires us to leverage the results of studies of assemblages from sites in the immediate area around a natural source. A recent study of thousands of artefacts from a single volcanic glass source in the Hawaiian Islands where material was also quarried informally suggests direct access should leave behind cortex on 25–50% of an assemblage [15]. As a corollary, we would expect all stages of reduction to be present, reflected in a large average artefact size.

Trade and exchange, either as informal down-the-line or formal long-distance movement, should leave different patterns. Material left behind from informal exchanges should show low frequency of cortex and decreased average size with distance from the source, as cores were reduced with each new owner. In contrast, formal trade and exchange should rarely leave behind cortex since quarrying reduction will occur at the time of collection to avoid the extra weight of unusable cortex. Clearly, raw material could be taken unaltered, but that would mean carrying, paddling, or sailing with more weight than was necessary. Alternatively, some source material may have subtle smooth cortex that could be mistaken for a fresh surface, and the incidence of raw material that has no cortex is unknown, but we suspect these are the exceptions rather than the rule. The average size of collections of imported material should be close to direct accessed raw material. We would expect this to be true even where imported material entered in to a local informal trade and exchange network after it was imported.

### Chemical Characterisation

X-ray fluorescence (XRF) and other techniques have been used to match obsidians to source through geochemistry in New Zealand archaeology for over 40 years (see [16] for a review). Recently, laboratory based use of inexpensive portable energy dispersive XRF, or pXRF, has proved an especially useful addition to these techniques [17]–[20]. In this study we used a BrukerAXS™ pXRF in the archaeology laboratories of the University of Otago. All samples were shot using optimal settings for ‘mid-z’ trace elements (Rb, Sr, Y, Zr, and Nb), specifically 40 kv and 8 microamps at a 300 second live time and with a filter (12milAl +1milTi+6milCu, or what the manufacture refers to as the ‘green’ filter). To examine lighter elements (Si, Ti, Al, Fe, Mn, Mg, Ca, Na, K), a second protocol was used that engaged the Bruker pXRF's vacuum, with the beam set to 15 kv and 45 microamps, but with no filter. For both settings, laboratory specific quantification protocols were created and applied. Linear regressions were based on nine pelletized international standards each shot three times for each of the two setting. Green filter linear regressions were improved by applying Speakman's [21] OB40 calibration to raw counts before the lab specific linear regression. A pelletized USGS basalt standard (BHVO-2) was run alongside samples as a quality check of precision and accuracy (Table 1), with an additional second standard (SRM-278) run on the vacuum setting to check the pXRF performance over a range of values (ppm) (see Document S1).

**Table 1:** Basalt Standard Chemistry.

BHVO-2	Ca	Fe	Rb	Sr	Zr
USGS recommended, ppm	82191	86030	9.8	389	172
Otago Lab, ppm	77578	86768	10.2	378	159
sd, ppm	211	72	2	18	7
RSD	0.3%	0.1%	19.5%	4.7%	4.1%

BHVO-2 standard was shot n = 43 at the green setting (40 kv, 8microamps) and n = 3 at the vacuum setting (15 kv, 15microamps). High relative standard deviation (RSD) in Rb is due a known problem of high variance in quantitative data when concentrations are low. These elements were used to assign obsidian artefacts to their likely geological source, see Document S1 for a full account of geochemistry.

We also looked closely at the assemblages and eliminated non-obsidians initially labelled in the field as ‘obsidian.’ These most commonly included dark coloured chert used by Maori in a similar fashion as obsidian, as well as unaltered dark coloured stones initially mistaken for artefacts. We note that South Island pitchstones, sometimes found among assemblages of ‘obsidian’ [18], were not found at these sites.

## Results

### Raw Material Used to Create Artefacts

**Figure 2:**
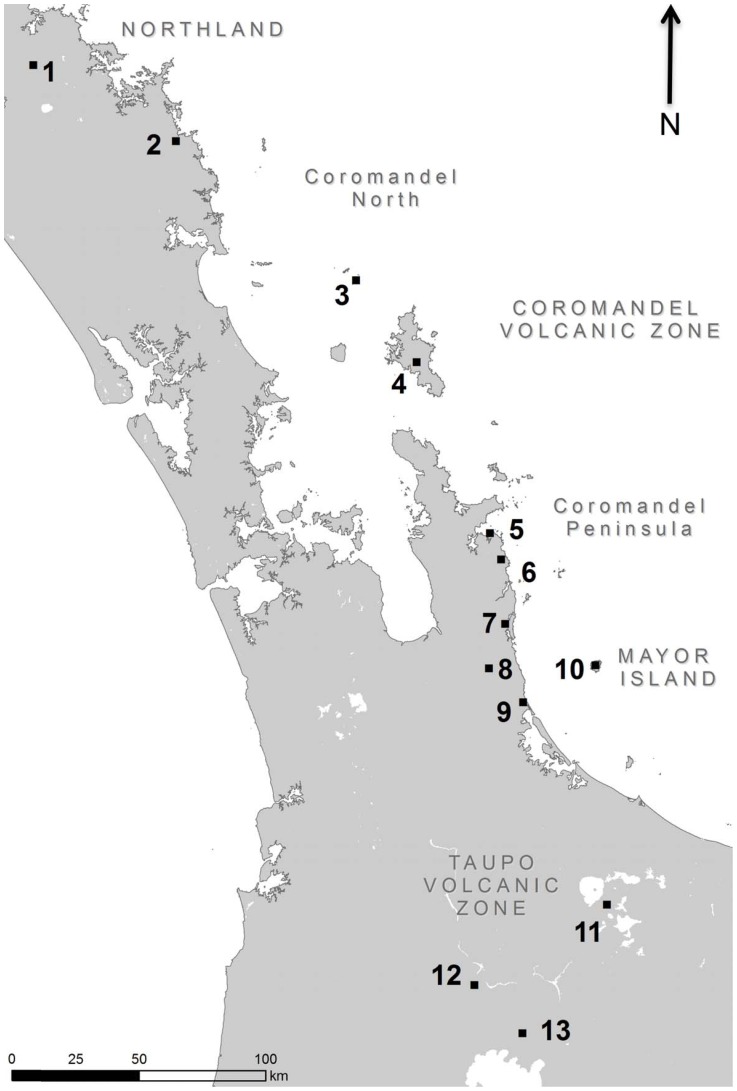
Natural Sources of North Island Obsidian.

New Zealand has four volcanic zones which have produced obsidians: Northland, the Coromandel Volcanic Zone (CVZ), Mayor Island, and the Taupo Volcanic Zone (TVZ) (Fig. 2). The CVZ can be broadly subdivided into natural sources of obsidian north of the Coromandel Peninsula (CVZ-N), and those on the peninsula itself (CVZ-CP) [22]. Across the whole of the North Island there are approximately two dozen different locations where archaeologists have posited that Maori could have obtained ‘flake-quality’ obsidian, however these collection areas rarely have obvious signs of quarrying outside of small discrete obsidian working areas (Table 2). It is more useful to think of these as source areas [16]. In this study we used geological reference material from the collections at the University of Otago that include examples of nearly all posited quarry locations, and certainly all of the sources identified in previous studies as having been definitively used to create stone tools [23].

**Table 2:** Geological Samples of New Zealand Obsidians (n = 70).

Map Key	Volcanic Zones	Source Name	Geological Samples
1	Northland	Kaeo	n = 2; GS464, GS560
2	Coromandel North (CVZ-N)	Huruiki	n = 10; GS167, GS171, GS195, GS234, GS237_8, GS238_3, GS257, GS361, GS364, GS518
3		Fanal Island	n = 1; GW255
4		Te Ahumata	n = 4, GS140, GS146, GS148, GS148_1
5	Coromandel Peninsula (CVZ-CP)	Cooks Bay	n = 5; GS546, GS591, GS601, GS610, GT844
		Hahei	n = 3; GT466_1, L21, UL1
6		Tairua	n = 4; GS629, GS631, GS632, GS639
7		Onemana	n = 1, Onemana-1
8		Maratoto	n = 1, GT847
9		Waihi	n = 2; GT841, GT843
10	Bay of Plenty	Mayor Island	n = 12; GS716, GS717, GS741, GS797, GS808, GS859, GS898, GT619, GT643, GT699, GT732, GT751
11	Taupo (TVZ)	Rotorua	n = 10; GS958, GS980, GS983, GT126, GT148, GT18, GT229, GT476, GT50, GT91
12		Maraetai	n = 4; GT279, GT282, GT288, GT304, GT793
		Ongaroto	n = 4; GT346, GT354, GT355, GT363
13		Taupo	n = 7; GT397, GT500, GT542, GT549, GT560, GT578, GT585

Sheppard et al. [20] have published a step-wise method to assign unknown New Zealand obsidian artefacts to source that begins by assigning artefacts to the two most chemically distinct groups – Mayor Island, and the Northland region source called Kaeo. The next step involves a hierarchical clustering analysis where artefacts are assigned source by end node membership. Misclassification is rare with this method, but in practice we have found it difficult to reproduce and there are inherently difficult sources to discriminate using the range of elements employed. For example, when we tested the Sheppard et al. [20] method on our geological samples we found relatively good results for the Huruiki source, but there was consistent mis-assignment of the Coromandel Peninsula and Taupo Volcanic Zone sources; a factor openly acknowledged by the authors. Further, the existing method was designed for assemblages without small artefacts. Small obsidian artefacts will however yield quantitative results that while imprecise, do follow a predictable trend in the ratio of elements, making it possible to match them to source. In this study we used pXRF to screen obsidian artefacts larger than the size of the x-ray aperture (3.5 mm diameter), and this included some pieces that likely produced quantitative results (ppm) lower than the actual concentration of elements because they were extremely thin.

**Figure 3:**
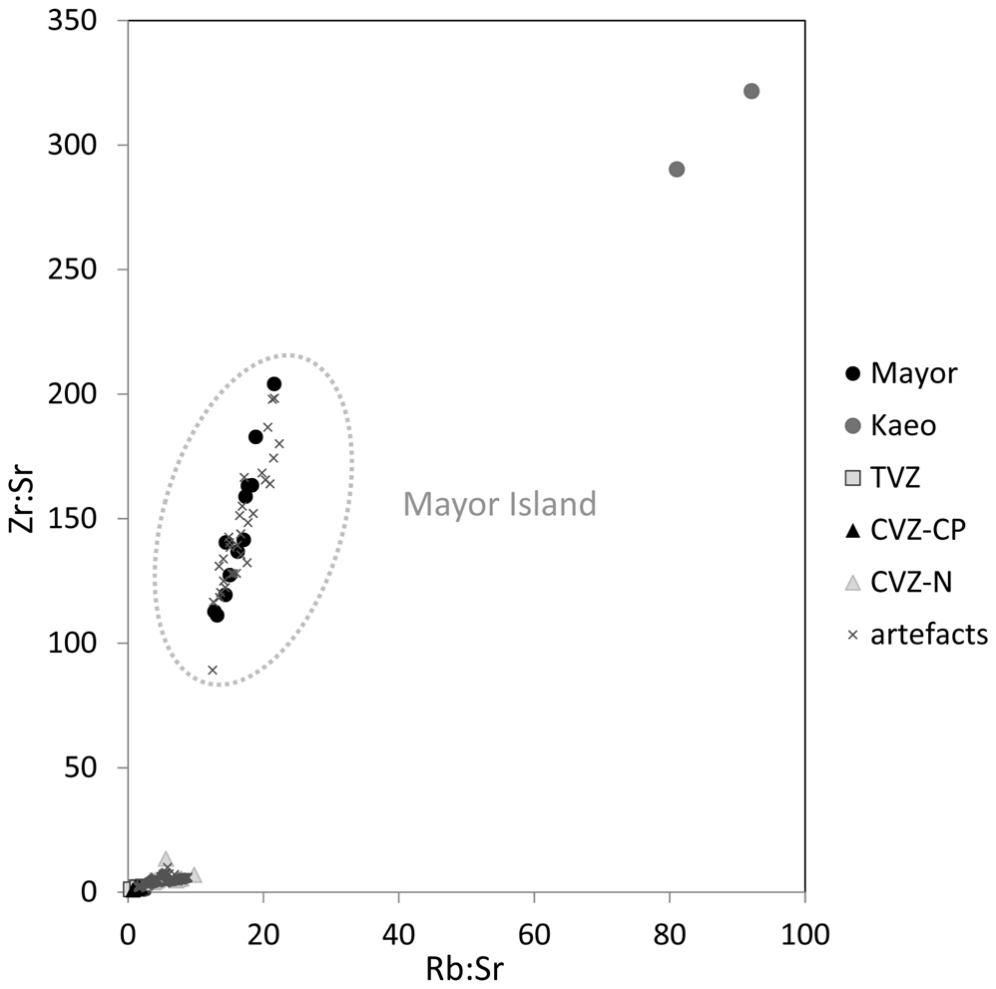
Artefacts Matched to the Mayor Island Source. Note that all ratios are on quantified data (ppm), not raw counts.

The method we used to assign artefacts to source here is also step-wise, but in this approach we wanted to define a series of steps that were easy to replicate, dealt with this known problem of overlapping ranges in the chemistry of CZV and TVZ sources, and was useful for small artefacts as well as larger ones. In the first stage, we use a bivariate plot of elements of rubidium (Rb) and zirconium (Zr) ratioed to strontium (Sr). This plot of Rb:Sr and Zr:Sr shows clear distinctions between Mayor Island, Kaeo, and all other obsidians (Fig. 3). In total, 30 artefacts were assigned to Mayor Island and none to the Kaeo source.

**Figure 4:**
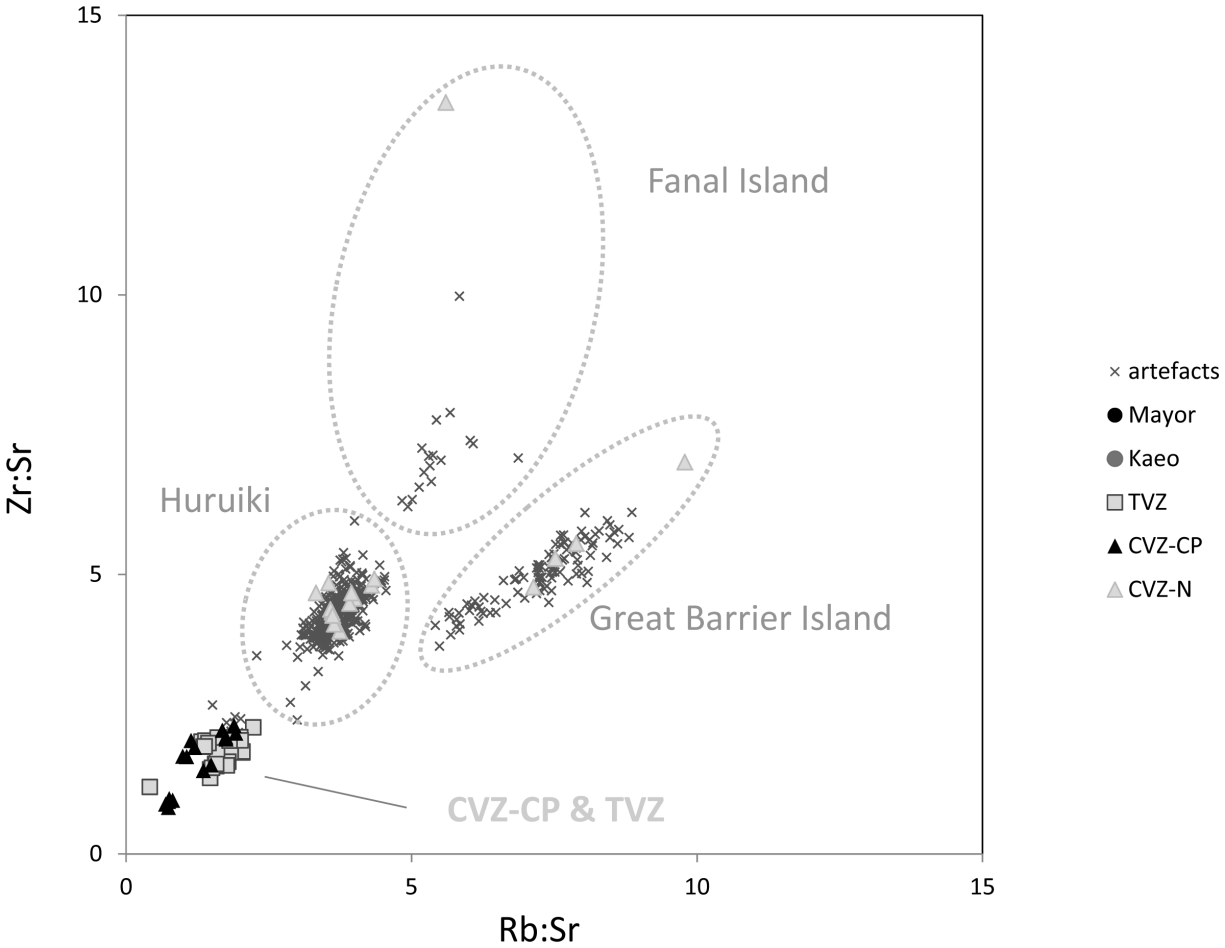
Artefacts Matched to Coromandel North Sources (Huruiki, Great Barrier Island, Fanal Island). Note that all ratios are on quantified data (ppm), not raw counts.

In the second step, this same plot was re-examined at a scale that allows us to distinguish between the three major CVZ-N sources located north of the Coromandel Peninsula (Great Barrier Island, Fanal Island, and Huruiki) with the remaining sources from the Coromandel Peninsula and Taupo Volcanic Zone muddled within the same cluster shown in the lower left (Fig. 4). Not surprisingly given how close these are to the sites examined here, the majority of artefacts fell within one of the three CVZ-N groups, with 97 from Huruiki, 101 from Great Barrier Island (Aotea), and 17 from Fanal Island. We note our geological sample of Fanal Island (Otago Lab identification, GW255; Field specimen identification P10233) has an unusually high Zr content; a factor noted in other geochemical analyses as suggesting it is not representative of the type of Fanal Island obsidian used by Maori.

**Figure 5:**
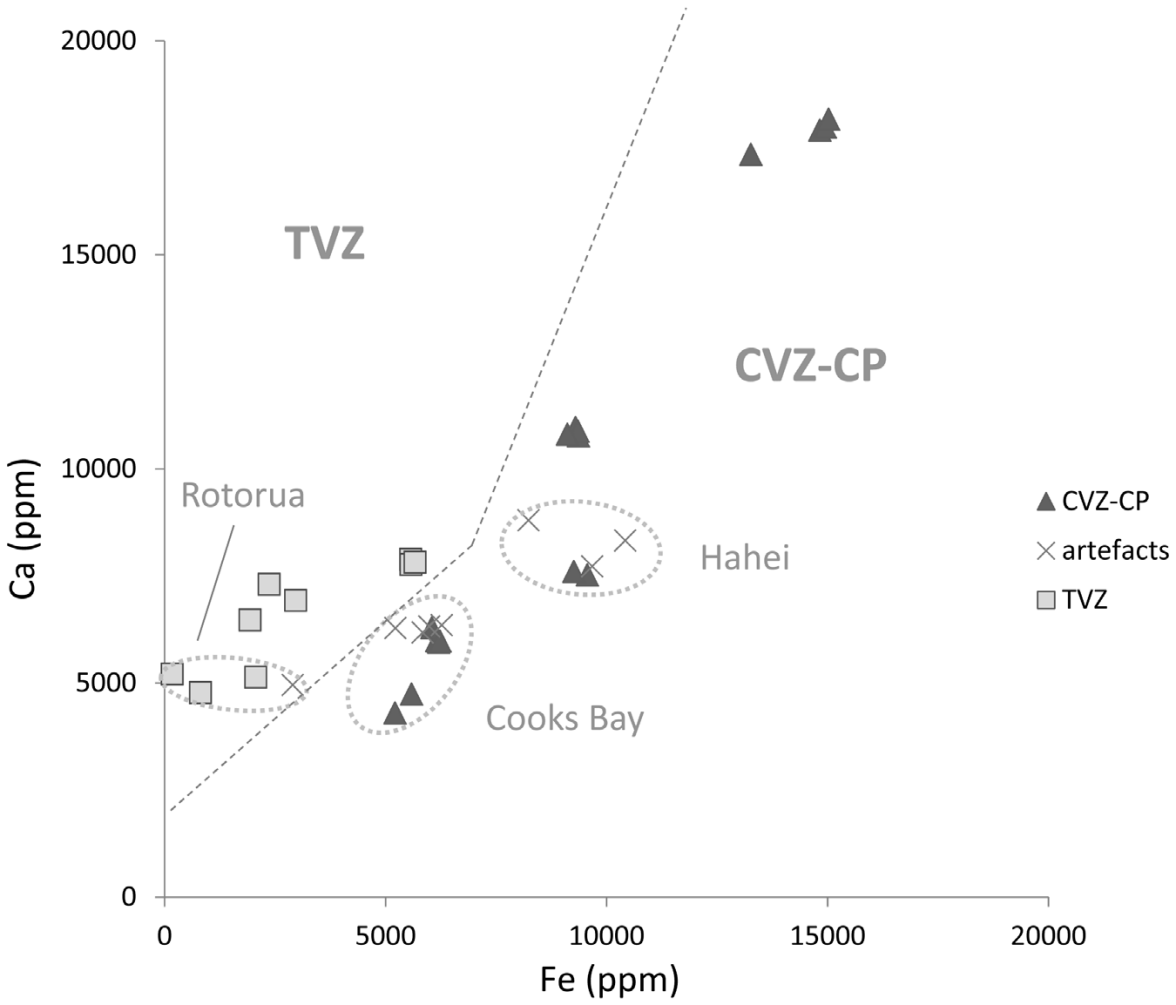
Artefacts Matched to Taupo Volcanic Zone (Rotorua Source) and Coromandel Peninsula (Cooks Bay/Hahei source). Cooks Bay and Hahei are chemically distinct from one another but treated as a single geographic source area.

For the last step, we turn to quantitative data from the vacuum setting, specifically the ratio of iron (Fe, ppm) to calcium (Ca, ppm) (Fig. 5). Since this step does not employ ratios to compensate for small-artefact readings, we would normally eliminate artefacts yielding readings that fall below a certain total count as not having sufficient data to be matched beyond this point. But in this case this was not necessary. Artefacts clearly fall in to a trend of low Ca:Fe for the TVZ material and high Ca:Fe for the CVZ-CP sources. It is possible to further identify which of the individual sources an artefact is likely from, however we note that this level of assignment comes with the caveat that more study of intra-volcanic zone chemical variation of obsidians is necessary to evaluate how confident we can be in this degree of detail. Most of the eight artefacts examined in this step match the Coromandal Peninsula (n = 7) - Cooks Beach (n = 4) and Hahei (n = 3) - and one from the Taupo Volcanic Zone is consistent with Rotorua (n = 1). Table 3 gives the final tally of artefacts assigned to source for each location. We should note that given that Cooks Beach and Hahei are geographically close to one another they are treated here as a single natural source area with two chemically distinct obsidians.

**Table 3:** Obsidian Artefacts Matched to Source.

Source	Helena Bay	Bream Head	Mt Wellington	Total
Huruiki (CVZ)	95	1	1	97
Fanal Island (CVZ)	-	12	5	17
Great Barrier Island (CVZ)	-	5	96	101
Mayor Island	-	15	15	30
Cooks Bay (CVZ)	-	-	4	4
Hahei (CVZ)	-	-	3	3
Rotorua (TVZ)	-	-	1	1
Total	95	33	125	253

As we might expect, obsidian found at Helena Bay comes exclusively from the Huruiki source. This was confirmed by chemically characterising a larger sample (n = 329) to determine if the large number of debitage from working material from the immediate vicinity of the site was masking evidence of obsidian coming from other sources. Again, all samples in this larger study match the Huruiki source.

At Bream Head, almost all material came from off-shore island sources, Mayor Island (46%), Fanal Island (36%), and Great Barrier Island (15%); with the closest mainland source accounting for a small amount (Huruiki, 3%). A recent pXRF study of obsidian from excavations near Bream Head (n = 17) also reported a dominance of these same sources, Mayor Island (35%), Great Barrier (35%), Fanal Island (12%), and few local mainland sources (Huruiki at 6%, Kaeo at 6%), and a Coromandel Peninsula source (Cooks Beach at 6%) [24]. Moore attempted assigning artefacts from another nearby site to source by visual assessment alone and reported a much higher proportion of Huruiki [25]. But when his estimates were verified by XRF, two out of the three artefacts assigned to Huruiki were found to in fact have come from Great Barrier Island.

At Mt. Wellington, the vast majority of artefacts came from these same off-shore islands, in this case Great Barrier Island (78%) was the dominant source followed by Mayor Island (12%), and Fanal Island (4%). The remainder are from the Coromandel Peninsula (6%), Rotorua in the Taupo Volcanic Zone (1%), and Huruiki (1%).

### Direct Access

Next, lithic collection strategies were assessed at the level of groups defined by source and site (e.g., Great Barrier Island obsidian found at Mt. Wellington). Only two groups are too small in number for lithic technology assessment, the single artefacts made of Rotorua and Huruiki obsidian found at Mt. Wellington. Since these represent examples of the furthest distant mainland sources found at the site, they are briefly discussed alongside evidence of long-distance formal trade.

**Figure 6:**
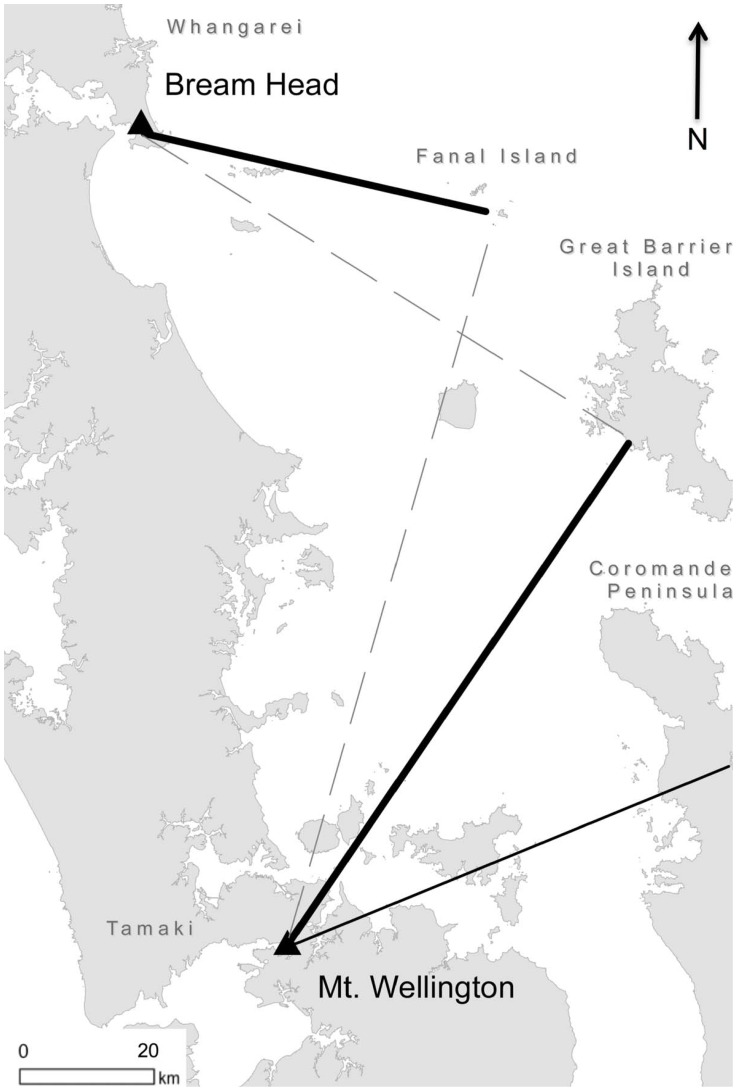
Obsidian Directly Accessed (solid) and Obtained by Informal Exchanges (dashed). Helena Bay site not shown since it is within several kilometers of the Huruiki source. Common sources accessed (thick lines) and those rarely accessed (thin line) are both shown.

**Figure 7:**
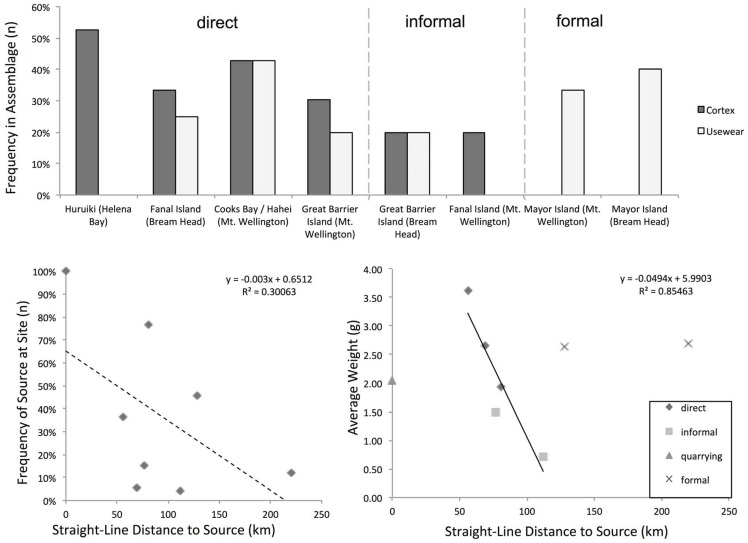
Collection Methods by Distance to Source. Directly accessed collections have larger average sizes, higher frequency of cortex (25-50%). Use wear is common outside of quarrying sites. Obsidian that was accessed by down-the-line informal exchanges is smaller on average; use wear is present. Obsidian from formal trades lacks cortex but use wear is common. The frequency of sources decreases with distance from the source but the trend is weak (lower, left; r^2^ = 0.30). However, if one limits to looking at weight vs. distance on directly accessed and informal exchange assemblages, the trend is much stronger (r^2^ = 0.86). The exceptions are quarry locations, where larger sized pieces are likely missing from assemblages having been taken away, and material that has been traded in which is far larger than one would expect relative to distance.

Of the material identified to source here, four groups fit the expectation of direct access, three of which are major contributors to the assemblages: Huruiki obsidian at Helena Bay, Fanal Island obsidian at Bream Head, and Great Barrier Island obsidian at Mt. Wellington (Fig. 6). In addition, Cooks Bay/Hahei obsidian found at Mt. Wellington, while only accounting for a small amount of the material found there, nonetheless fits the criteria of having larger than average size and a high frequency of cortex (Fig. 7; Table 4). In this case, we note that assemblages with +1.75 g average weight and more than 25% of artefacts with cortex are included in this group, which we feel is appropriate given the broad range of assemblages examined, but note that these are only rough estimates. We note that there is strong evidence for usewear in each group, except Helena Bay. This confirms the initial assessment that Helena Bay deposits primarily reflect quarry reduction unlike the other sites where obsidian is deposited from a mix of core reduction and post-use discard. Another commonality between these groups is they represent the closest natural sources to sites, with the exception of Bream Head where a large proportion of material is from the far distant Mayor Island source. Nonetheless, directly accessed Fanal Island obsidian is the most common of the local sourced obsidian (i.e., 2/3 of local artefacts come from Fanal Island). Overall, direct access accounts for the majority of artefacts examined here, even putting aside quarrying at Helen Bay, 71% of the artefacts (112 out of 158) were likely the result of people accessing sources on Fanal Island, Great Barrier Island, and Cooks Bay/Hahei.

**Table 4:** Summary of Methods Used to Collect Obsidians.

Source	Site Location	Straight line distance (km)	Frequency	Ave (g)	Cortex	Use	Method
Huruiki	Helena Bay	0	100%	2.05	53%	0%	direct (quarrying)
Fanal Island	Bream Head	56	36%	3.62	33%	25%	direct
Cooks Bay-Hahei	Mt. Wellington	69	6%	2.66	43%	43%	direct
Great Barrier Island	Mt. Wellington	81	77%	1.94	30%	20%	direct
Great Barrier Island	Bream Head	77	15%	1.49	20%	20%	informal
Fanal Island	Mt. Wellington	112	4%	0.72	20%	0%	informal
Mayor Island	Mt. Wellington	128	45%	2.65	0%	33%	formal
Mayor Island	Bream Head	220	12%	2.70	0%	40%	formal

### Informal Trade-and-Exchange

Two groups fall in to the expected range for informal trade and exchange: Great Barrier obsidian found at Bream Head, and Fanal Island obsidian found at Mt. Wellington (Fig. 6). Artefacts in these assemblages show 20% frequency of cortex and are on average smaller than material gained from direct access and, although we only have these two examples, there is a decrease in average size with distance (Fig. 7). These again are rough metrics but are nonetheless consistent with material circulating without having first been reduced at the source for transport, however, not as much cortex as would be present if it were directly accessed. Overall, each accounts for a relatively small amount of the total site assemblages (15% and 4%, at Bream Head and Mt. Wellington, respectively). Again, putting the deposits associated with quarrying at Helena Bay aside, informal exchange accounts for just 6% of artefacts (10 out of 158).

### Formal Trade-and-Exchange

The only groups that fit the expectations for formal trade involve Mayor Island obsidian. To illustrate how unusual Mayor Island collections are, in a relative sense, we present several metrics of the reductive process and working of obsidian (Fig. 7). For example, if we consider the frequency of cortex and useware, we find that among the group representing direct quarrying, Huruiki obsidian found at Helena Bay, about 50% of artefacts have cortex and there are no obvious signs of useware; facts consistent with an assemblage representing quarrying. At sites further away from sources, there is generally a mix of cortex and useware. Only Mayor Island obsidian shows a healthy amount of useware but no cortex. This fact could point to it having arriving already slightly reduced, either as cobbles turned in to blocks without cortex, or directly mined from natural outcrops without a weathered surface; more systematic study is necessary to demonstrate which is the case.

**Figure 8:**
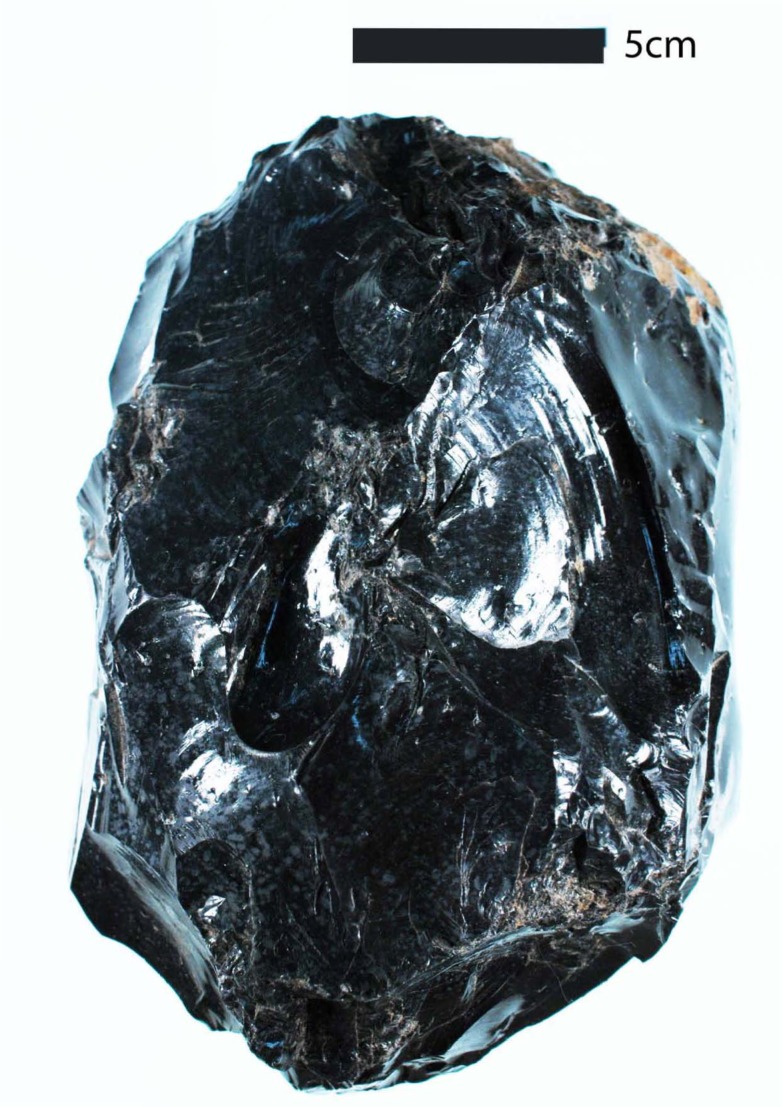
Large Obsidian Core. This 2.5

Archaeologists have noted that the shape of fall-off curves created by obsidian distance decay, i.e., the general decrease in frequency of obsidian with greater distance from a source, holds a great deal of potential information about ancient economies, but the interpretation of these must be approached carefully [26]–[31]. For example, a simple linear regression based on frequency and distance, while following an expected decline with distance, is a superficial description that does not account for the variation observed (r^2^ = 0.30). However, if we consider weight instead of frequency, and use lithic technology assessments of assemblages to limit the model to just those that already assessed to likely reflect directly accessed and informally exchanged groups, we find a clear linear decay with distance (r^2^ = 0.86); exactly what one would expect if the weight of material is a factor in people's decision making. What is more important here are the ‘outliers.’ We suspect Helena Bay Huruiki obsidian is smaller than expected relative to this trend because quarrying reduction represents the first working of a cobble, with the largest workable core pieces having been carried away from the site, and thus subtracted from the assemblage (Fig. 8). Mayor Island obsidian, however, is much larger than one would expect and there are a number of plausible explanations for this, but given the other information we have about these assemblages, we believe the most parsimonious explanation is that this reflects relatively large pieces of obsidian having been transported from directly in long-distance exchanges.

### Classifying Obsidian as ‘Local’ and ‘Non-Local’

**Figure 9:**
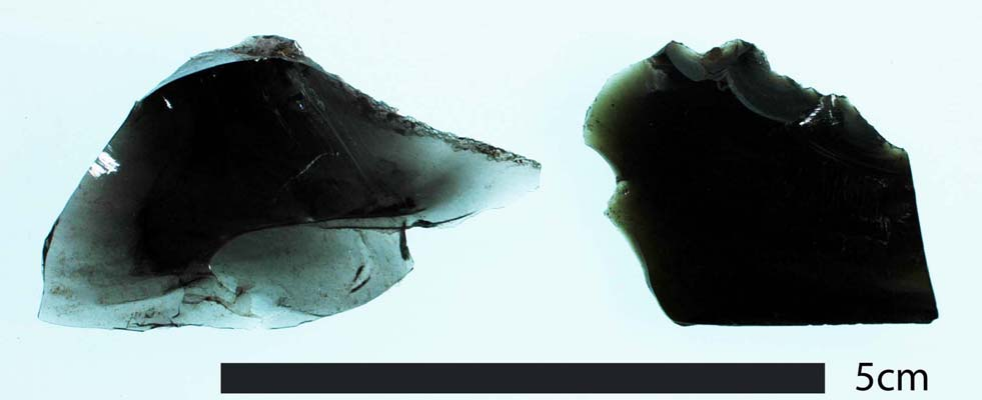
New Zealand's Grey and Green Obsidians. On left is an example of a grey obsidian artefact from Great Barrier Island (AR4032a), right is a green obsidian from Mayor Island (AR4005); both artefacts were recovered from Mt. Wellington.

Before we move on to discuss these results, we would like to take a moment and briefly address the question, in this part of New Zealand, is Mayor Island a ‘non-local’ or ‘local’ source? As sourcing studies have matured, archaeologists continue to improve on subjective terms like ‘long distance trade,’ by considering the time, effort, and mode of transport involved (e.g., [15]). But, while spatial and logistical analyses are important, we believe an equally important question in a country with so many natural sources is how would one know a particular piece of obsidian was from the immediate area, defined as the area one could complete a round-trip to in a single day either on foot or with the aid of a canoe? For example, single artefacts made from Huruiki (AR4020b) and Rotorua (AR4010) obsidians were found at Mt. Wellington. Morphologically speaking, in terms of colour, size, and form, they fall within the range of other artefacts found at the site (i.e., both are grey; AR4020a is a small tool, 0.31 g; and AR4010 is a large piece of shatter, 2.91 g). However, unlike material from local grey obsidian sources, Mayor Island obsidian is a distinctive green colour (Fig. 9). The Northland region's Kaeo obsidian source is also green, but as Moore [32] has argued, the trained eye can sometimes distinguish between the two. More importantly, regardless if a piece of green obsidian was from Northland (Kaeo) or the Bay of Plenty (Mayor Island), green obsidian in this part of New Zealand, especially Tāmaki, would have been immediately recognisable as distinct from local grey obsidian.

In some cases, an additional factor that may have clearly marked Mayor Island obsidian as distinct from other commonly used sources is its unusually large size. The maximum size of naturally occurring boulders and angular-to-subangular blocks of the obsidian sources discussed here have been reported in the +50 cm to 20 cm diameter range (Huruiki, ca. 20 cm [12]; Fanal Island, +50 cm, [33]; Great Barrier Island, ca. 40 cm, [34]). Like these sources, Mayor Island obsidian can also be collected as cobbles and boulders in this same size range. But, it is also home to thick flows exposed in “twin obsidian selvages” [35] that ring the island. Therefore, it is possible for one to mine out massively large pieces that would have been immediately recognisable as not occurring naturally within our study area.

## Discussion

The results of this study show direct access was commonplace, accounting for perhaps 70% of obsidian discarded at sites, and not surprisingly, all of the obsidian recovered from quarrying reduction at the Huruiki source. Long-distance formal trades and exchanges appear to be more common than informal down-the-line movement of material, at 19% and 6% respectively. However, appreciating what these results mean requires us to consider the costs and benefits of these choices with regard to Maori cultural values, specifically the importance of materialising social ties through resource access and the social obligations that arise in trade and exchange. Lastly, we would like to briefly address how we see this research inter-connects with the large and growing body of obsidian studies by archaeologists.

First, the discovery of commonplace direct access would appear to be at odds with the high concentration of fortifications built during the Late Period; why with such a great deal of material evidence for warfare would people choose to travel to neighbouring off-shore islands to collect raw material? One simplistic answer would be the mode of transport involved: people preferred to obtain obsidian by sea travel. The extremely low frequency of the closest mainland sources certainly would suggest overland foot travel may have been viewed as a much less attractive means of collecting obsidian. But, since coastal fortifications are common on both the mainland and off-shore islands, it is not the case that sea routes were unmonitored or less defended when compared with land routes. Therefore, the common theme of marine transportation, while interesting, in our view does not hold explanatory weight.

A better explanation has to do with materialising family and tribal connections between the Tāmaki region and Great Barrier Island, and the Whangarei region and Fanal Island, respectively. Davidson [10] notes that, the residents of Mt. Wellington were, “closely related people [to the residents of Great Barrier Island], some of whom lived both on Great Barrier Island and at Tāmaki (Graeme Murdoch, pers. comm. 2010).” In the Whangarei region, the Ngatiwai tribe also has strong ties to Great Barrier Island, but at a greater genealogical distance. In the period represented here, there was probably a more immediate connection with people living on off-shore islands north of Great Barrier, such as the Hen and Chicken Islands group (Marotere) and Fanal and Burgess Islands group (Mokohinau). Therefore, the more important common theme here is that these visits represented two-way efforts by communities to maintain a social connection through their common ancestry (*whakapapa*). Resident island communities who held customary guardianship (*kaitiakitanga*) over obsidian resources could have rejected the authority (*mana*) of their mainland kin and prohibited obsidian collection (*rahui*), but instead appear to have chosen to regard them with open access and hospitality (*manaaki*). This also appears to have sometimes been the case with regard to Coromandel Peninsula and Tāmaki tribes; but the more important point here is there was a persistent clear connection between Tāmaki and Great Barrier, and Whangarei and Fanal, that is evident in the archaeological record.

Second, the discovery of long-distance trade and exchange is not unexpected given the well documented movement of greenstone from the South Island to the North Island in the Late Period. At this stage, finished objects made from greenstone are well documented in assemblages of Late Period North Island sites [36], [37]. What does beg for explanation is why we have a material that people otherwise have regular direct access to being imported from a great distance away, and why so few down-the-line exchanges with neighbours? One simple explanation accounts for these trends: Mayor Island obsidian was preferred over other types of obsidian in formal exchanges and trades because it was immediately recognisable as non-local. In an exchange, a block of grey obsidian, even one that was from a great distance away, would look identical to local obsidian, including a source that the recipient had inherited rights to. Even with the above caveats regarding Maori concepts of ownership, you can't ‘give’ someone something they already ‘own,’ and even the appearance of doing so might insult to the recipient's authority (*mana*). Thus, green obsidian that is clearly from outside Tāmaki or Whangarei, may have been a practical solution to fit to the larger traditional framework of Maori values. Future research should focus on variability in Mayor Island obsidian found in deposits from across the Late Period as representing the use of this material in exchanges that had meaning embedded in local cultural practices, rather than a pattern that can be understood from a purely materialist perspective.

Finally, this study comes at a time when we are seeing a marked upswing in obsidian studies [38] and as spatial analyses have become more grounded in anthropologically informed models [39]. However this is, intellectually speaking, a well-travelled path beginning with Renfrew's [26], [28] ground breaking use of obsidian to reconstruct ancient economic systems, followed by Hodder's [40] call for a more substantivist approach to exchange in terms of “social obligations, status, and power,” and a torrent of work that has exposed the complexity of using this class of material to reconstruct human history. In this study, we have leveraged a materialist need (i.e., the need for raw material) against the rich direct historical information we have regarding Maori society in general and the specific relevant tribal histories. It is our wish that this study encourage similar ones in Pacific Island archaeology where we can apply a direct historical approach to learn how contact period socio-economic systems developed over time, and thus provide archaeologists working in other areas without the benefit of direct historical information a dynamic analogy through which to view similar datasets.

## Conclusions

It would appear that Maori living on the North Island's north-eastern coast in the Late Period (1500–1769 A.D.) primarily obtained their obsidian through direct access to source areas on off-shore islands. A small but significant amount of obsidian continued to arrive from the Bay of Plenty's Mayor Island as whole blocks, perhaps presented as gifts in formal exchanges. Down-the-line trade and exchange does not account for much of the obsidian represented in archaeological deposits. This is unsurprising given the ready access people had to collect obsidian directly, and the preference for recognisably non-local obsidian in formal exchanges, gifts, or trades.

These results run counter to the expectation that direct access to natural sources was less attractive than other means to supply oneself with obsidian during a period with outward archaeological signals of high endemic warfare (i.e., earthwork fortifications). Rather, Maori who lived during this period appear to have worked to maintain kin networks across regions, and these connections were materialised through obsidian collection. This is not to say that warfare was not part of the history of this era, it most certainly was. However, archaeologists have an obligation to look beyond just the source of obsidian, or the frequencies of sources at sites, and take a hard look at the lithic technology represented in assemblages and reflect on what those patterns tell us about how people maintained social connections despite the threat of violent conflict.

## Supporting Information

Document S1 **Geochemistry for Geological and Archaeological Samples of New Zealand Obsidians.**
(XLS)
